# Cross Protection against Influenza A Virus by Yeast-Expressed Heterologous Tandem Repeat M2 Extracellular Proteins

**DOI:** 10.1371/journal.pone.0137822

**Published:** 2015-09-14

**Authors:** Yu-Na Lee, Min-Chul Kim, Young-Tae Lee, Hye Suk Hwang, Jongsang Lee, Cheol Kim, Sang-Moo Kang

**Affiliations:** 1 Center for Inflammation, Immunity & Infection, Institute for Biomedical Sciences, Georgia State University, Atlanta, Georgia 30303, United States of America; 2 Animal and Plant Quarantine Agency, Anyang, Gyeonggi-do, South Korea; 3 BEAMS Biotechnology Co. Ltd., Seongnam, Gyeonggi-do, South Korea; The University of Chicago, UNITED STATES

## Abstract

The influenza M2 ectodomain (M2e) is well conserved across human influenza A subtypes, but there are few residue changes among avian and swine origin influenza A viruses. We expressed a tandem repeat construct of heterologous M2e sequences (M2e5x) derived from human, swine, and avian origin influenza A viruses using the yeast expression system. Intramuscular immunization of mice with AS04-adjuvanted M2e5x protein vaccines was effective in inducing M2e-specific antibodies reactive to M2e peptide and native M2 proteins on the infected cells with human, swine, or avian influenza virus, mucosal and systemic memory cellular immune responses, and cross-protection against H3N2 virus. Importantly, M2e5x immune sera were found to confer protection against different subtypes of H1N1 and H5N1 influenza A viruses in naïve mice. Also, M2e5x-immune complexes of virus-infected cells stimulated macrophages to secrete cytokines via Fc receptors, indicating a possible mechanism of protection. The present study provides evidence that M2e5x proteins produced in yeast cells could be developed as a potential universal influenza vaccine.

## Introduction

Current influenza vaccines based on influenza virus surface glycoprotein hemagglutinin (HA) antigens are effective if the predicted vaccine strains and circulating viruses are to be well-matched. However, it is difficult to predict potential pandemic strains. The emergence of the swine-origin 2009 H1N1 pandemic virus explicitly demonstrated how current influenza vaccination was not effective in controlling a new pandemic [1]. In addition, avian influenza viruses such as H5, H7, and H9 subtypes continue to circulate in nature, raising serious concerns for their possibility of sustained human-to-human transmission [2, 3]. Therefore, development of effective vaccines conferring a broad range of cross protection is considered to be of high priority.

The extracellular domain of M2 (M2e), which contains 24 amino acids, is highly conserved among human influenza viruses although there are few residue changes among avian and swine influenza viruses [4, 5]. Therefore, M2e-based vaccines have been investigated as an attractive candidate for a universal influenza vaccine with broad-spectrum protection [6–8]. Previous studies have shown that immunogenicity of native M2e is poor, but it can be increased by using multimeric forms of M2e, fusion of M2e-to highly immunogenic carrier or applying with adjuvants [9–17]. However, many of these adjuvants such as cholera toxin and Freund’s adjuvant would not be appropriate for human use due to their potential side effects [18].

Yeast has been considered as a promising organism to express viral vaccines due to its several advantages when compared with insect or mammalian cells in terms of large scale vaccine production and a short period of production time provides new vaccines for rapid response to influenza pandemic threats. Moreover, yeast-based expression platforms offer additional attractive features including low operating costs, simple culture conditions and no viral and endotoxin contamination. Yeast-based expression of recombinant vaccines against human hepatitis B virus and human papilloma virus was licensed for human use [19, 20].

Adjuvant System 04 (AS04) combining of toll-like receptor 4 ligand MPL (3-O-desacyl-4’-monophosphoryl lipid A) and aluminum hydroxide (Alum) was approved for human vaccines such as hepatitis B and papilloma virus vaccines [21, 22]. In the present study, we investigated the immunogenicity of M2e tandem repeat (M2e5x) soluble proteins expressed in yeast (*Pichia pastoris*) with AS04. In addition, humoral and cellular immunogenicity as well as breadth of cross-protective were examined.

## Materials and Methods

### Preparation of M2e5x proteins

A DNA fragment of M2e5x construct was cloned into the pPIC9 vector (Life technology, NY, USA) for secretory expression in the yeast through the use of the α-factor mating secretion signal. The recombinant plasmid was first linearized with the SalI enzyme and used to transform *P*. *pastoris* strain GS115, phenotype Mut+ (methanol utilization plus) by electroporation, in accordance with the manufacturer’s instructions (Life Technologies). *P*. *pastoris* transformants were inoculated into BMGY medium (1% yeast extract, 2% peptone, 1.34% YNB, 1% glycerol, 100 mM potassium phosphate, pH 6.0) and incubated at 30°C for 48 h. For induction of the M2e5x protein, yeast cell culture media were changed with BMMY medium (the same components as those of BMGY with glycerol replaced by 0.5% methanol) and methanol was added subsequently.

M2e5x recombinant proteins were purified by two-step chromatography consisted of hydrophobic interaction chromatography (HIC) and anion exchange chromatography (AEX). Phenyl-Sepharose FF was used as HIC for capturing chromatography of M2e5x from the *P*. *pastoris* (GS115) fermentation broth (S1 Fig). Fermentation broth was adjusted to be 1.5M ammonium sulfate (A/S) by directly adding powder A/S and adsorbed onto Phenyl-Sepharose (GE Healthcare, USA). M2e5x proteins were eluted by 0.7M A/S in 0.5 x PBS (pH7.4) after washing with 5 column volume (CV) of wash buffer (1.5M A/S in 0.5 x PBS). The eluted active pool of Phenyl-Sepharose was applied to Sephadex G25 (GE Healthcare, USA) column in order for desalting and buffer exchange with 1 x PBS (pH7.4). Q-Sepharose FF (GE Healthcare, USA) used to the second purification step as polishing chromatography. Q-Sepharose column was equilibrated with 5 CV of equilibrium buffer (0.05M NaCl in 0.5 x PBS). And then, the desalted M2e5x proteins were reconditioned to be equilibrium buffer condition and loaded onto Q-Sepharose. Q-Sepharose column was washed with 5 CV of wash buffer (0.1M NaCl in 0.5 x PBS) and eluted the active pool with the buffer (0.15M NaCl in 0.5 x PBS). The Q-Sepharose active pool of M2e5x proteins was exchanged with 1x PBS (pH7.4) as a storage buffer through the Sephadex G25 gel-filtration and sterilized with 0.22 μm filter for preparation of purified M2e5x proteins.

### Viruses, cells and reagents

Embryonated chicken eggs were used to grow A/California/04/2009 (A/CA04, H1N1; a gift from Dr. Richard Webby) and reassortant A/Vietnam/1203/2004 (A/VN1203, rgH5N1 containing H5 HA with polybasic residues removed, N1 NA from A/VN1203 and 6 internal genes from A/PR/8/1934) [23] and A/Mandarin Duck/Korea/PSC24-24/2010 (A/PSC24-24, rgH5N1 containing HA with polybasic residues removed, NA and M genes from A/PSC24-24, and the remaining backbone genes from A/PR/8/1934 virus) as previously described [23]. Purified inactivated viruses were produced by treating formalin at a final concentration of 1:4000 (v/v) as described previously [24]. Madin-Darby canine kidney (MDCK) cells were purchased from ATCC and cultured in Dulbecco’s Modification of Eagle’s Medium with 10% fetal bovine serum. Four sets of M2e peptide (a2-20), human type-SLLTEVETPIRNEWGSRSN, swine type-SLLTEVETPTRSEWESRSS, avian type I-SLLTEVETPTRNEWESRSS, and avian type II-SLLTEVETLTRNGWGCRCS were synthesized (GenScript, Piscataway, NJ), and used in this study. Mouse anti-influenza virus M2 monoclonal antibody (mAb) 14C2 (Abcam, Cambridge, MA) was used for detection of M2e5x protein by western blot.

### Immunization and challenge

Groups of nine female BALB/c mice (6 to 8 weeks old, Harlan Laboratories) were immunized intramuscularly with 20 μg of M2e5x protein (total protein) or mixed with AS04 adjuvant (5 μg MPL plus 50 μg Alum) at week 0, 4, and 8. Blood samples were obtained three weeks after each immunization. Mice were challenged intranasally four weeks after third immunization with a lethal dose (4.5 x LD_50_) of A/Phil influenza virus and body weight loss and survival rates were daily monitored for 14 days post-infection (p.i.). If mice show body weight loss up to the endpoint 25% compared to the age-matched control animal, then mice are euthanized immediately. Even before the mice reach the endpoint, the mice that show clinical signs of illness such as ruffled hair coat and/or difficult breathing are humanely euthanized to avoid the pain. The methods of euthanasia used in the study include isoflurane and carbon dioxide, and death is confirmed by cervical dislocation. Animals are anesthetized using isoflurane inhalation before the distressful procedures to relieve the pain or suffering. All animal experiments presented in this study were approved by the Georgia State University IACUC review boards (A11026).

### Assays for antibody responses

The antibody level specific to M2e was evaluated by enzyme-linked immunosorbent assay (ELISA) using 96-well immunosorbent plates were coated with 2 μg/ml of different types of M2e peptides as previously described [25]. Influenza virus-infected MDCK cells were fixed with 10% buffered formalin and used to determine antibody levels binding to M2e expressed on infected cell surfaces. Sera were serially diluted, added in duplicate and incubated for 1.5 h at 37°C. Following washing again horseradish peroxidase (HRP)-labeled goat anti-mouse IgG, IgG1, and IgG2a (Southern Biotechnology, Birmingham, AL) were added to each well (1:2,000), and incubated for additional 2 h at 37°C. Substrate solutions containing tetramethybenzidine peroxidase substrate (Sigma-Aldrich, St. Louis, Mo) were used to develop color and the absorbance at 450 nm was measured using an ELISA reader (Bio-Rad).

### Preparation of BALF, virus titration and cytokine assay

Challenged mice were euthanized at 5 days p.i. For bronchoalveolar lavage fluids (BALF), the diaphragms were dissected to allow free lung expansion and the lungs were lavaged three times by slowly instilling 1 ml of PBS then gently aspirating. Each lung was homogenized and centrifuged at 1400 × g at 4°C for 10 min. The supernatants were inoculated into the allantoic cavity of 9–11-day-old embryonated eggs to determine lung viral titers as described in detail previously [26]. After 72 hr of incubation at 37°C, the eggs were chilled, and allantoic fluid harvested from each egg was tested for hemagglutinin activity. The infectivity of the virus in the supernatant was determined from the 50% egg infectious dose (EID_50_). Cytokines in BALF were assayed with ELISA kits in duplicate against a standard curve according to the manufacturers’ instructions (eBioscience, San Diego, CA).

### Determination of M2e-specific antibody secreting cell and T cell responses

On day 5 p.i., spleen and bone marrow cells were isolated from infected mice for single cell preparations that were subsequently cultured in 96-well plates coated with M2e5x protein using a modified method as previously reported [23]. IgG antibody levels specific to M2e peptide in the culture supernatants were determined by ELISA as described above. Interferon (IFN)-γ-secreting cell spots were determined on Multi-screen 96 well plates (Millipore, Billerica, MA) coated with IFN-γ cytokine capture antibodies (BD Biosciences, San Diego, CA) as previously described [27]. Briefly, spleen and lung cells were isolated from challenged mice and cultured on the plate with M2e peptide (2 μg/ml). After 36 h incubation, the plates were further incubated with biotinylated rat anti-mouse IFN-γ (BD Biosciences) followed by HRP-conjugated streptavidin solution and substrate stable DAB (3, 3-Diaminobenzidine, Invitrogen) to develop color. The numbers of color spots representing IFN-γ-secreting T cells were counted using an ELISpot reader (BioSys, Miami, FL). To evaluate intracellular cytokine production, lung cells were stimulated with 5 μg/ml of M2 peptide (SLLTEVETPIRNEWGSRSN) at 37°C for 5 h, and were surface stained with fluorophore-labeled surface markers (CD45, CD4, CD8, and Fc blockers), and then were made permeable using the Cytofix/Cytoperm kit (BD Biosciences). Intracellular cytokines were revealed by staining the cells with anti-IFN-γ-antibodies as described previously [28]. Stained cells were analyzed using LSRFortessa (BD Biosciences) and FlowJo software (Tree Star Inc.).

### *In vivo* protection assay of immune sera

To assess cross-protective efficacy of immune sera *in vivo*, immune or naïve sera after heat inactivation (56°C, 30 min) were mixed with an equal volume of virus A/VN1203 (rgH5N1), A/CA04 (H1N1), or A/PSC24-24 (rgH5N1 virus containing an avian type M2e) at a lethal dose (6 x LD_50_) and incubated at room temperature for 30 min as described in detail previously [29, 30]. Naive BALB/c mice were inoculated with a mixture of virus and sera, and were monitored daily for their body weight changes and survival rates for 14 days p.i.

### Preparation of bone marrow derived macrophages (BMDMs) and cytokine assays

Bone marrow cells from FcRγ deficient mice (FcR γ^-/-^ encoded by *Fcer1g* on the BALB/c genetic background, Taconic farm) and wild type (WT) BALB/c mice were cultured in sterile plastic petri dish with 4 x 10^5^ cells in DMEM complete medium including with 25 ng of recombinant mouse macrophage-colony stimulating factor for 7 days to enrich macrophages. MDCK cells (10^7^) were infected with 10 multiplicity of infection of A/Phil influenza virus and incubated for 15 h at 37°C. Cells were washed once with PBS before gentle dissolution with a cell scraper and resuspended in 1 ml of PBS. The immunized or naïve sera were inactivated at 56°C for 30 min and preabsorbed with MDCK cells to avoid nonspecific binding to MDCK cells. The mixtures of MDCK cell pellets and 200 μl of the immune or naïve sera (10-fold diluted) were incubated with BMDMs (4 × 10^5^ cells/ml) for 2 days. TNF-α, IL-6, and IL-12 cytokines were determined in the BMDM culture supernatants using ELISA as described above.

### Statistical analysis

Statistical analyses were done using GraphPad Prism software. Data are presented as means ± error of the mean (SEM). Differences between groups were analyzed by 1-way analysis of variance (ANOVA) or 2-way ANOVA where appropriate. P-values less than 0.05 were regarded as significant.

## Results

### Characterization of soluble protein containing tandem repeat M2e

**Fig 1:**
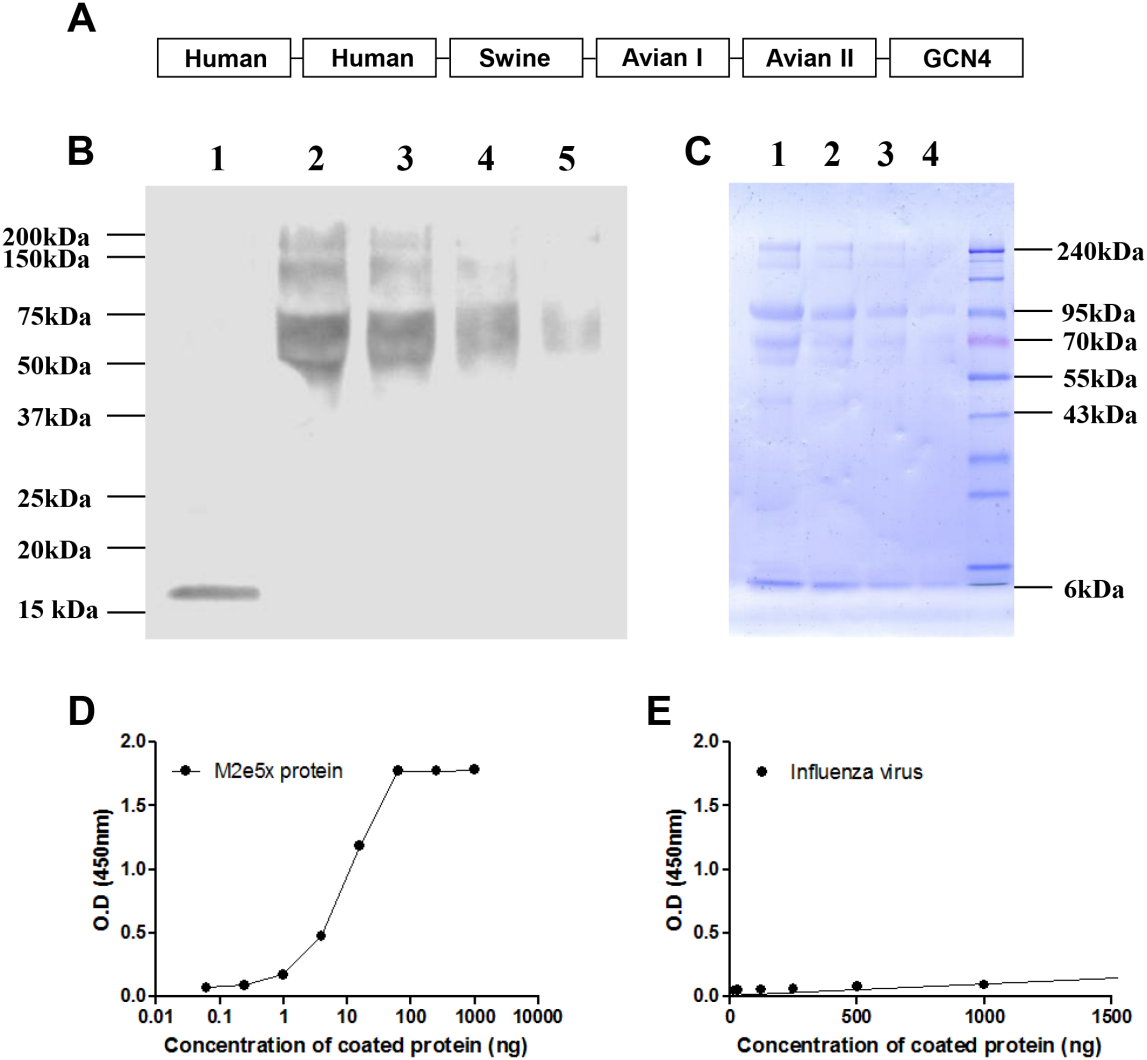
Characterization of tandem repeat M2e5x protein. (A) Structures of M2e5x protein. Human: Human influenza A type M2e, Swine: Swine influenza A type M2e, Avian I: Avian influenza A type I M2e, Avian II: Avian influenza A type II M2e,-: linker. (B) Western blot of M2e5x protein using mouse anti-M2 monoclonal antibody (14C2 mAb). Lane 1: influenza A/Philippines/2/82 (H3N2) virus (40 μg). Lane 2–4: M2e5x protein (10 μg, 5 μg, 2 μg, 1 μg). All samples were run under reduced conditions. (C) SDS-PAGE analysis of M2e5x protein. Lane 1–4: M2e5x protein (40 μg, 20 μg, 10 μg, 5 μg). (D) ELISA reactivity of 14C2 mAb to M2e5x proteins. (E) ELISA reactivity of 14C2 mAb to influenza A/Philippines/2/82 (H3N2) virus.

A soluble protein containing five copies of influenza virus M2e sequences from human type (2x), swine type (1x), major avian type I (1x) and minor avian type II (1x), and a tetramer-stabilizing leucine zipper GCN4 domain [31] was designed to counteract the variation of M2e epitopes (Fig 1A). With the aim of inducing more antibodies that can confer protection against human influenza A viruses mainly, two copies of human type M2e sequence were included. The identity and integrity of the soluble protein were estimated by Western blot analysis (Fig 1B) and ELISA using 14C2 monoclonal antibody (mAb) specific for M2e (Fig 1D and 1E). The results of high reactivity to 14C2 mAb confirmed the presence of M2e epitopes in the purified M2e5x proteins. Western blot using 14C2 mAb shows the bands with a molecular weight of approximately 75 kDa, unlike the VLP containing similar tandem repeat of 37 kDa in our previous study, which possibly be due to the incorporation of signal sequence and multimerization of M2e5x proteins. The purity of M2e5x protein was approximately 60% as analyzed by SDS-PAGE (Fig 1C). Compared with that of A/Phil (H3N2) virus, the reactivity of 14C2 mAb to M2e5x proteins was significantly higher (Fig 1D and 1E). Since 14C2 mAb has been shown to recognize protective epitopes in M2 [32, 33], the results provide evidence that protective epitopes of M2e would be presented in an immunogenic form in M2e5x proteins produced in yeast cells.

### AS04 adjuvanted M2e5x proteins induce M2e-specific antibody responses

**Fig 2:**
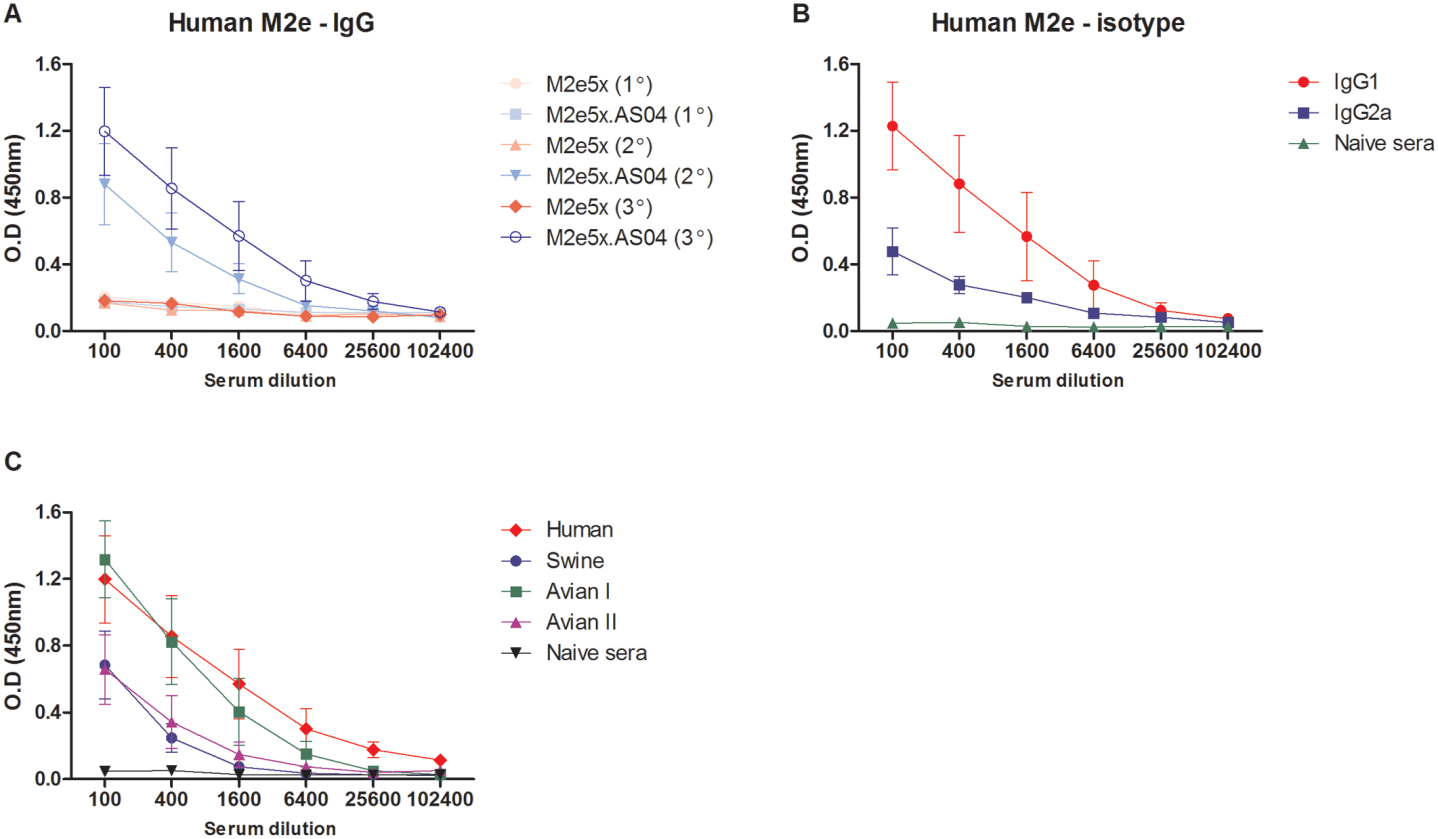
AS04-adjuvanted M2e5x protein vaccination induces M2e-specific IgG and IgG isotype antibody responses. BALB/c mice (*n* = 9) were immunized with 20 μg of M2e5x protein alone (M2e5x) or mixed with adjuvant AS04 (M2e5x.AS04). Blood samples were collected at 3 weeks after each immunization. (A) Total IgG antibody. IgG was detected by using human type M2e peptide as an ELISA-coating antigen. (B) M2e-specific IgG isotype responses of the M2e5x.AS04 group after 3rd immunization. (C) IgG antibody responses reactive to M2e peptide antigens derived from human, swine, or avian influenza A viruses. IgG antibodies specific to M2e were measured in 3rd immune sera from the M2e5x.AS04 group using human, swine, avian I, or avian II type peptide as a coating antigen. Sera were serially diluted and ELISA was performed for serum antibodies specific for M2e peptides. Error bars indicates mean ± SEM.

To evaluate the immunogenicity of M2e5x protein vaccines alone (M2e5x) or formulated with AS04 (M2e5x.AS04), groups of mice were intramuscularly immunized and antibody responses in sera were determined by ELISA using a human type M2e peptide antigen (Fig 2A and 2B). At 3 weeks after boosting, M2e-specific antibodies were detected at significant levels in the M2e5x.AS04 group (Fig 2A). After the 3rd immunization, antibodies specific for M2e were observed at significantly increased levels, over 4-fold higher compared with those observed after boost in the M2e5x.AS04 group. Immunization with M2e5x protein alone or in the presence of alum induced only marginal levels of M2e-specific antibodies. The anti-M2e IgG isotypes were determined by ELISA after 3rd immunization (Fig 2B). The main isotype antibody by M2e5x.AS04 immunization was IgG1, and to a lesser extent IgG2a. Protein vaccination typically induces a predominantly Th2 response. Therefore, Th2-baised antigenic characteristics of M2e5x proteins seem to be responsible for IgG isotype switching even if these proteins are formulated in AS04 adjuvant.

We investigated whether immune sera from the M2e5x.AS04 group would be reactive with M2e peptide sequences derived from swine or avian influenza viruses (Fig 2C). Immune sera collected from the M2e5x.AS04 group showed significantly high levels of antibody reactivity to human and avian type I M2e peptides. Serum antibodies by M2e5x.AS04 immunization were also found to be substantially reactive to swine and avian type II M2e peptides although their levels were lower than the reactivity to human and avian type I M2e peptides. These results indicate that M2e5x protein formulated with AS04 is immunogenic, inducing antibodies recognizing different types of M2e antigens.

### AS04 adjuvanted M2e5x protein immunization improves protection against influenza virus

**Fig 3:**
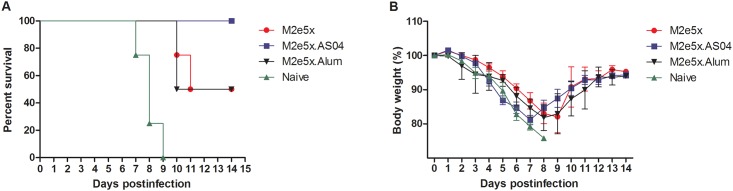
Vaccination with AS04-adjuvanted M2e5x protein induces improved protection. Groups of BALB/c mice (*n* = 4) were challenged with a lethal dose of A/Philippines/2/82 (H3N2) virus at 4 weeks after 3rd vaccination. Survival rates (A) and body weight (B) were monitored for 14 days. Error bars indicates mean ± SEM. The data are representative out of two independent experiments.

To compare the efficacy of protection, groups of mice that were intramuscularly immunized with M2e5x protein alone or formulated with AS04 were challenged with a lethal dose (4.5 x LD_50_) of A/Phil virus at 4 weeks after second boost (Fig 3). All naive control mice lost over 25% in body weight and died or had to be euthanized. The survival rates of the mice vaccinated with M2e5x protein alone and formulated with alum or AS04 were 50%, 50% and 100%, respectively (Fig 3A). The groups of mice with M2e5x alone, M2e5x.alum and M2e5x.AS04 showed a substantial loss of approximately 18% in body weight post challenge (Fig 3B). Interestingly, the M2e5x.AS04 group showed a trend of higher weight loss compared to the M2e5x group at early time points (4 to 7 dpi.), but there was no significant difference. Nonetheless, the M2e5x.AS04 group recovered body weight faster than the M2e5x group after 7 day p.i.

**Fig 4:**
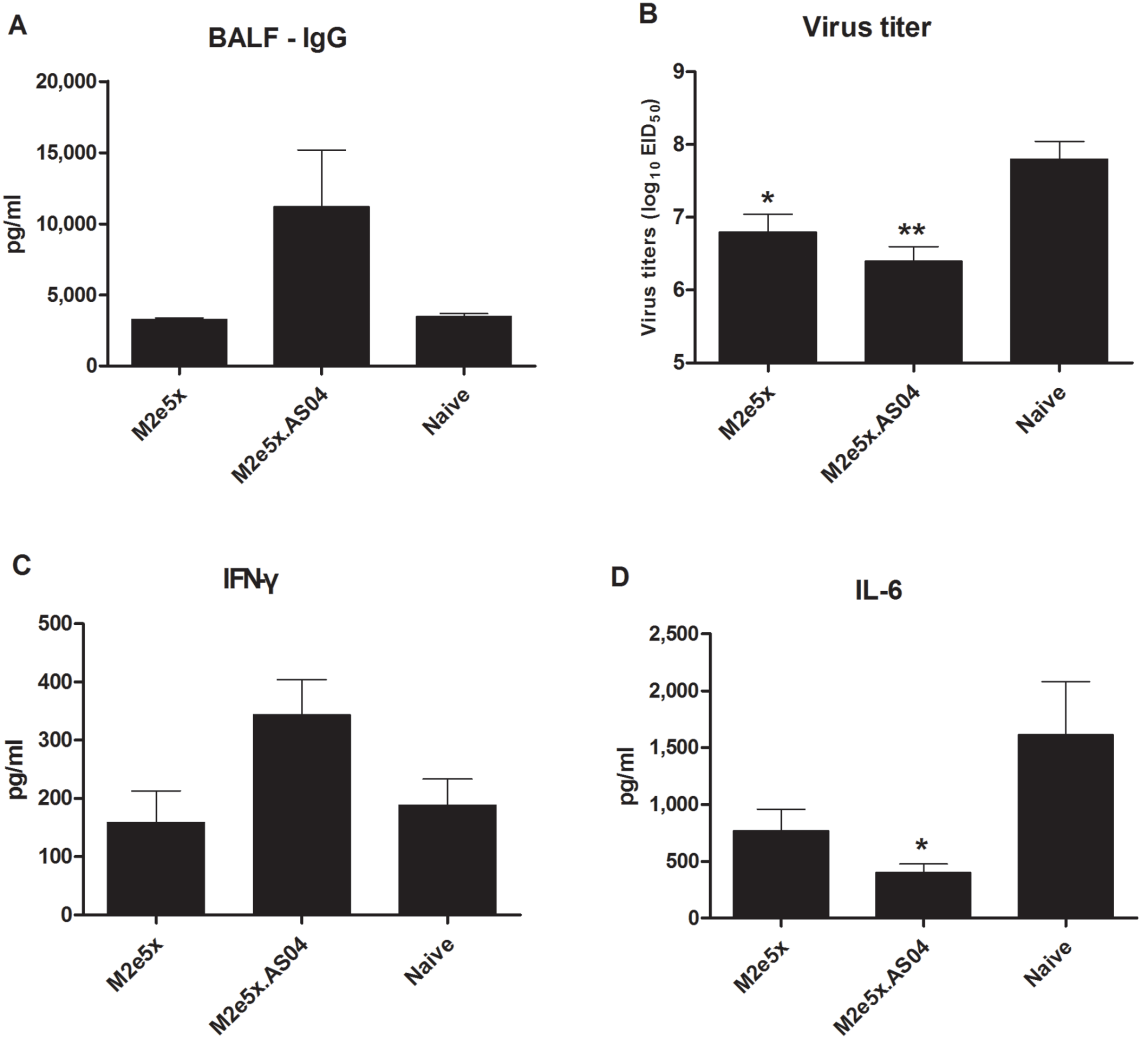
Antibody responses, virus titers, and inflammatory cytokine levels in BALF and lungs after challenge. Levels of IgG antibodies were determined in samples collected from mice at day 5 p.i. with A/Philippines/2/82 (H3N2) virus (*n* = 5). (A) IgG antibody levels in BALF. The antibody level was determined by ELISA using M2e peptide as a coating antigen. Lung viral titers (B), IFN-γ (C), and IL-6 (D) cytokines in BALF were determined at day 5 p.i. Lung viral titers were determined by an egg infection assay. IFN-γ and IL-6 were determined by a cytokine ELISA. Data represent mean ± SEM. Statistical significances were determined by 1-way ANOVA. Asterisks indicate significant differences (*p < 0.05 and **p < 0.01) compared with the results in the naïve group. ns, not significant.

M2e-specific IgG antibody responses in BALF were determined at day 5 after challenge with H3N2 virus (Fig 4A). M2e-specific IgG antibody concentrations in BALF from the M2e5x.AS04 group were approximately 3.2-fold higher than those from the M2e5x alone and naïve mice at day 5 p.i., but there was no significant difference. The M2e5x.AS04 (*p* < 0.01, Fig 4B) and M2e5x (*p* < 0.05) groups showed significantly lower lung viral titers compared with those in naïve control groups at day 5 p.i. There was no statistical difference between the titers in M2e5X with AS04 and M2e5X without AS04. The lowest lung viral titer in the M2e5x.AS04 group indicates a good correlation with 100% survival rate.

The levels of IFN-γ in BALF were found to be approximately 1.8-fold higher from the group of mice vaccinated with M2e5x.AS04 compared to those in the M2e5x protein alone and naïve groups at day 5 p.i. (Fig 4C). By contrast, a reverse pattern was observed with IL-6 inflammatory cytokine. Levels of IL-6 were observed at significantly lower in BALF from mice immunized with M2e5x.AS04 (*p* < 0.05) than those of naive mice, IL-6 inflammatory responses in lungs were induced probably due to high viral replication after challenge with A/Phil virus (Fig 4D). We did not observe a statistical significance in lung IL-6 levels between M2e5X with AS04 and M2e5X without AS04 groups.

### Enhancement of cytokine and antibody secreting cell responses by M2e5x protein vaccination with AS04

**Fig 5:**
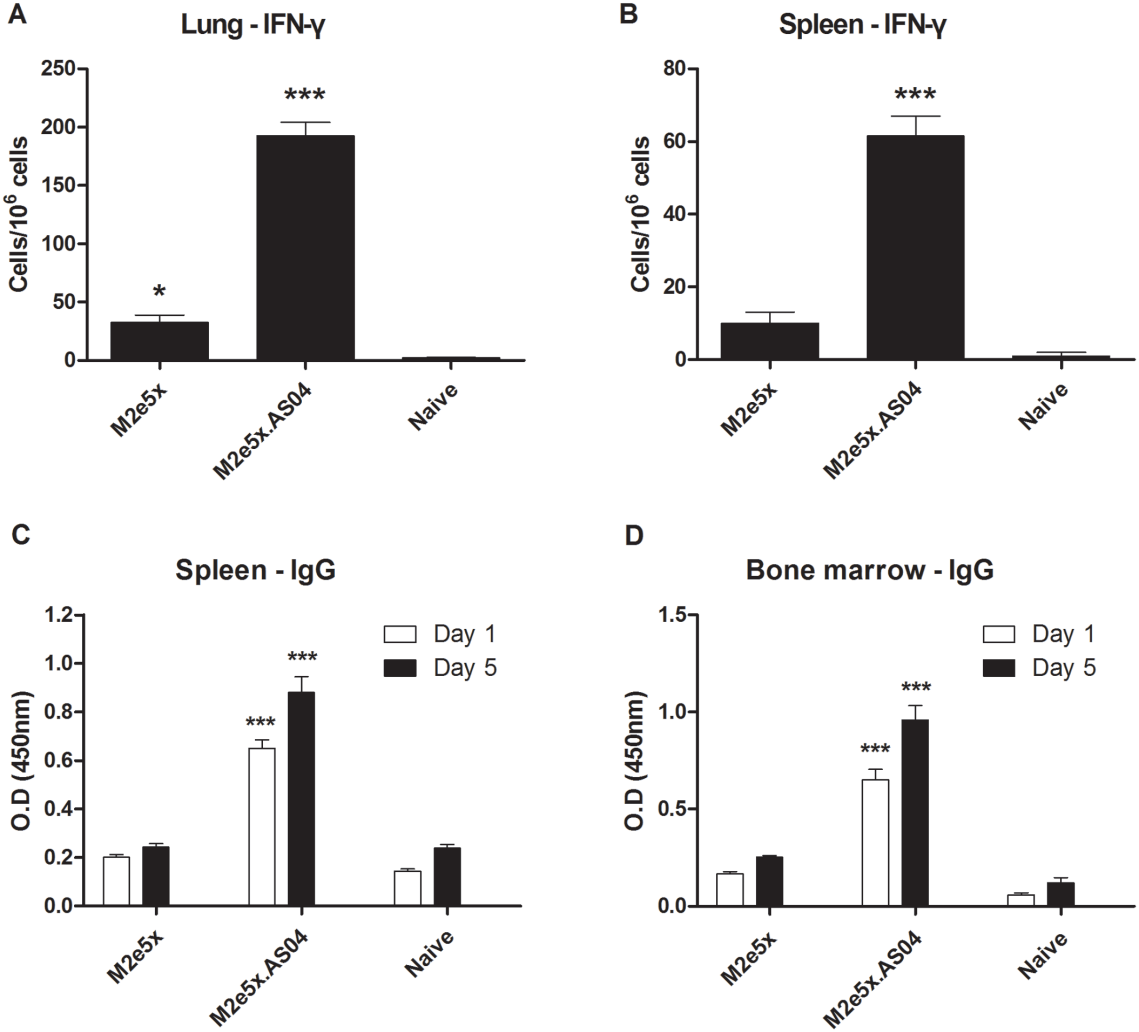
AS04-adjuvanted M2e5x protein vaccination is effective in inducing antibody-secreting cell or recall cellular immune responses. (A) IFN-γ-secreting cells in lungs. (B) IFN-γ-secreting cells in spleens. Lung cells and splenocytes were isolated from mice at day 5 p.i. (*n* = 5). Cytokine-producing cell spots were counted by ELISPOT reader. Spleen (C) and bone marrow cells (D) were isolated from mice at day 5 p.i. and were incubated in the presence of M2e5x protein coated on the culture plates for *in vitro* stimulation. Culture supernatants were harvested after 1 or 5 days of culture. M2e-specific IgG levels were determined by ELISA. Data represent mean ± SEM. Statistical significances were determined by 1-way ANOVA. Asterisks indicate significant differences (*p < 0.05 and ***p < 0.001) compared with the results in the naïve group.

To determine effector T cell responses, we measured IFN-γ-producing lung and spleen cell spots after *in vitro* stimulation with M2e peptide (Fig 5A and 5B). The spot numbers of IFN-γ–secreting cells were detected at a significantly higher level in lungs from mice in the M2e5x.AS04 group than those from the M2e5x group and naïve group (*p* < 0.001, Fig 5A). Interestingly, the M2e5x group showed substantial levels of IFN-γ–secreting cells in lungs which were significantly higher than those of naïve mice (*p* < 0.05). Thus, effector IFN-γ–producing T cell responses might have contributed to increasing the survival rate. Moreover, significantly higher levels of IFN-γ–secreting cells were observed in spleens from M2e5x.AS04-immunized mice compared to those from M2e5x protein alone and naïve mice (*p* < 0.001, Fig 5B). To investigate the role of CD4^+^ and CD8^+^ T cells producing IFN-γ in M2e-mediated protection, we measured IFN-γ-producing lung cells after *in vitro* stimulation with M2e peptide (S2 Fig). IFN-γ-producing M2e-specific CD4^+^ and CD8^+^ T cells were induced at significant levels in the M2e5x.AS04 group compared with those in the Naïve group (*p* < 0.01).

In order to evaluate M2e-specific antibody secreting cell responses, splenocytes and bone marrow cells were collected at day 5 p.i. and cultured *in vitro* for 1 and 5 days. Significantly higher levels of IgG antibodies specific to M2e were detected in the supernatants of splenocytes (Fig 5C) and bone marrow cell (Fig 5D) from the M2e5x.AS04 group than those from naïve mice (*p* < 0.001). These results indicate that vaccination with AS04-adjuvanted M2e5x proteins can induce the generation of memory B cells in spleens as well as plasma cells in bone marrow which can differentiate into antibody secreting cells upon influenza virus infection.

### Immune sera of M2e5x protein vaccination with AS04 confer improved cross-protection

**Fig 6:**
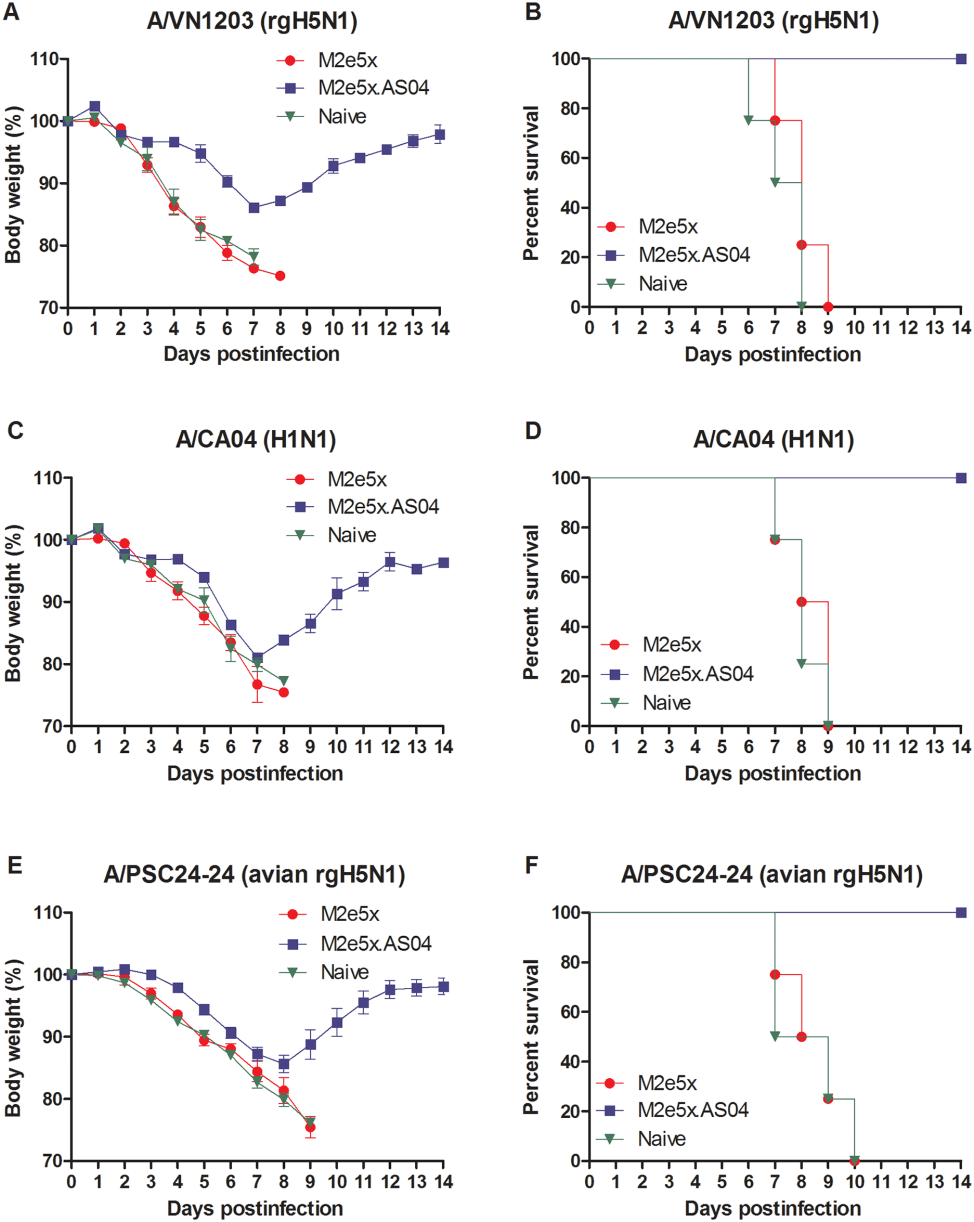
Immune sera of M2e5x protein with AS04 confer heterosubtypic cross-protection against H1N1 and H5N1 viruses. Mice (*n* = 4) were intranasally infected with a lethal dose of influenza virus mixed with immune sera (1x) or naive sera (1x). Immune sera collected from vaccinated mice after 3rd immunization were incubated with influenza viruses, reassortants A/Vietnam/1203/2004 (A/VN1203, rgH5N1) (A, B), A/California/04/2009 (A/CA04, H1N1) (C, D), and /Mandarin Duck/Korea/PSC24-24/2010 (A/PSC24-24, rgH5N1) (E, F). Body weight (A, C, E) and survival rates (B, D, F) were monitored for 14 days.

We further evaluated whether sera with higher binding activity to M2e would confer broad cross-protection against influenza viruses with a different type of M2e. Naive mice were inoculated with a mixture of immune sera and different strains of influenza A virus. Sera from M2e5x immune or naïve mice did not confer any protection to naïve mice, respectively (Fig 6). In contrast, immune sera from mice vaccinated with M2e5x.AS04 showed 100% protection to naïve mice that were infected with A/VN1203 (Fig 6A and 6B) or A/CA04 with swine type M2e (Fig 6C and 6D) or A/PSC24-24 with avian type I M2e (Fig 6E and 6F). Moderate levels of morbidity (14–19% weight loss) depending on the virus strain used for infection were observed in protected mice. Nonetheless, these results support the evidence that AS04-adjuvanted M2e5x protein vaccination can induce antibody responses which confer protective immunity to multiple H1N1 and H5N1 subtypes of influenza viruses with different types of M2e.

### Cytokine production is impaired in FcRγ deficient macrophages after stimulation with immune complexes

**Fig 7:**
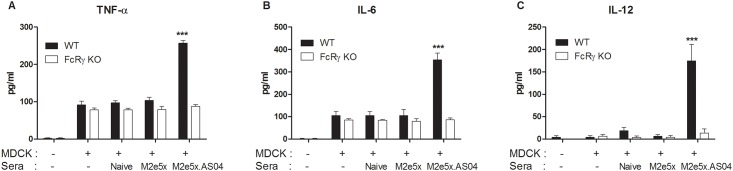
Cytokine production in FcRγ-deficient macrophages by immune complexes. (A-C) MDCK cells were infected with A/Philippines/2/82 (H3N2) virus overnight and subsequently incubated with naïve or M2e5x.AS04-immune sera. The mixtures of MDCK cell pellets and immune or naïve sera were incubated for 2 days with BMDMs (4×10^5^ cells/ml) from BALB/c wild type (WT) or FcRγ deficient mice. TNF-α (A), IL-6 (B), and IL-12 (C) was determined by a cytokine ELISA. Data represent mean ± SEM.

Macrophages are known to contribute to phagocytosis of influenza virus-infected cells [34] and to protection against influenza virus by M2e antibodies via Fc receptors [12, 35–37]. Here, we assessed the contribution of FcRγ to cytokine production by activating BMDMs through immune complexes of influenza-infected MDCK cells and M2e immune sera. Interestingly, we found that the levels of TNF-α (Fig 7A), IL-6 (Fig 7B), and IL-12 (Fig 7C) in the supernatants from WT BMDMs incubated with the mixture of influenza A virus-infected cells and M2e5x.AS04-immunized sera were significantly higher than those from FcRγ^-/-^ BMDMs (*p* < 0.001). By contrast, the levels of the cytokines in the supernatants from FcRγ^-/-^ BMDMs stimulated with M2e sera-infected cell immune complex were similar with those from naïve or M2e5x only immune sera-immune complex or virus-infected cells only. These results provide the evidence that the Fc γ receptor may play a role in inducing cytokines by stimulating macrophages with immune complexes and thus in M2e antibody-mediated protection against influenza virus infection.

## Discussion

Although the first 9 aa of M2e are well conserved among diverse influenza A viruses, there are few aa differences in the 15 remaining aa residues among the human, swine and avian influenza viruses [6, 38]. It has been reported that the cross-reactivity of antibodies induced by M2e-carrier constructs based on consensus M2e sequences with complete Freund’s adjuvant were determined against several M2e peptides, and no or low cross-reactivity was observed with avian M2e peptides that differed by 3 to 4 aa from the consensus sequence [11, 39]. In order to overcome heterogeneity of M2e among influenza A viruses, we expressed in yeast cells a genetically designed vaccine construct with tandem repeat of M2e consisting of representative M2e sequences from human, swine, and avian influenza viruses. Moreover, immune sera to M2e5x.AS04 vaccination were found to confer cross protection against H1 and H5 subtype influenza viruses even if these viruses have swine and avian M2e sequences respectively. Previously it was shown that heterologous tandem repeat M2e5x VLPs confer broader and stronger immunity than homologous human influenza M2e VLP [36]. In the present study, we found that yeast cell-expressed heterologous M2e5x protein vaccines adjuvanted with AS04 can be immunogenic and confer broad spectrum of cross protection against diverse subtypes of influenza A viruses. Previously, we developed a vaccine construct with tandem repeat of M2e, which was expressed in a membrane-anchored form using the recombinant baculovirus expression system and presented on the enveloped virus-like particle (M2e5x VLP) that was effective in inducing M2e-specific antibodies and cross-protection regardless of influenza virus subtypes [36]. Presenting M2e5x on the VLP platform seemed to be a superior immunogen using a lower concentration (10μg of total proteins) compared to the yeast-expressed M2e5x protein (20μg of total proteins). A disadvantage is that baculovirus is difficult to be separated from influenza VLPs due to the similar size and density. A certain level of viral contaminants is likely to be co-purified during VLP preparations [40–42]. Although there is evidence that these baculovirus contaminants are safe for immunization [43, 44], a low level of contaminant in VLPs can be a regulatory hurdle in translating a vaccine candidate into a commercial product. The advantage of AS04 adjuvanted-M2e5x protein vaccines is that the yeast-based expression system of recombinant vaccines against human hepatitis B virus and human papilloma virus was licensed for human use [19, 20]. Therefore, our findings demonstrated that a strategy to design tandem repeat of multiple heterologous M2e sequences and to express it in yeast cells can be a promising approach for broader cross-protective vaccines against influenza A viruses.

M2e in itself is poorly immunogenic, due to its small size, and several strategies have been attempted to improve its immunogenicity including the fusion of M2e peptides with carrier proteins or vehicles or formulation in adjuvants such as complete or incomplete Freund’s adjuvant [11], cholera toxin subunits [45], heat-labile endotoxin [46, 47] or liposome based cationic adjuvant [48]. Despite their ability to stimulate immune response, these adjuvants would not be licensed for human use because of potential severe side effects. AS04 is one of the new generation adjuvants recently licensed for use in humans [49]. Previously, we demonstrated that insect cell-expressed M2e5x VLP adjuvanted with AS04 was more effective than M2e5x VLP alone in conferring protection after lethal influenza virus challenge in C57BL/6 mice [28]. Consistent with previous findings, yeast cell-produced M2e5x protein adjuvanted with AS04 was more effective than M2e5x protein alone in protecting vaccinated mice against lung viral replication as well as in increasing survival rates after lethal influenza virus challenge. Although AS04-adjuvanted M2e5x proteins did not modulate the immune response toward a Th1-biased profile, these formulations improved the immunogenicity of M2e5x proteins. In addition, we observed significant levels of splenocytes and lung cells secreting IFN-γ cytokine, a typical Th1 cellular response in the M2e5x.AS04-adjuvanted group. An MPL-based adjuvant has been found to specifically activate local follicular helper T cells that are thought to be directly associated with the generation of B cell memory [50, 51]. Moreover, alum provides signals required for the generation of long-lived memory cells, whereas MPL promotes IFN-γ production by antigen-specific CD4^+^ T cells and enhance cytotoxic CD8^+^ T cell differentiation [52, 53]. Concordantly, we observed higher frequencies of memory B cells and IFN-γ-producing T cells following M2e5x protein vaccination with AS04. Results in this study support that AS04 adjuvant effectively enhances the immunogenicity and anamnestic responses of M2e5x protein vaccines.

It is important to note that M2e immunity was not able to prevent severe weight loss due to lethal influenza virus infection as shown in the M2e5x.AS04 group. The less capability of M2e immunity to protect against weight loss was reported in previous studies demonstrating that M2e immune mice displayed severe weight loss after lethal challenge [14, 15, 48, 54]. Protective efficacy by immunization with M2e-hepatitis B core conjugate vaccine was much weaker than HA based inactivated virus vaccination [55]. An alternative approach to improve both protective efficacy and the breadth of cross protection might be to supplement HA-based vaccines with conserved M2e vaccines. In support of this approach, supplementing inactivated virus or split vaccines with M2e conserved epitope vaccines was shown to improve protective efficacy of preventing weight loss as well as broadening the cross protection [12, 56–58].

Cellular immune responses are believed to play an important role in controlling influenza infection, and in improving recovery and survival rates during later phases of infection. Several studies have shown that M2e-specific T cells can mediate protection against influenza infection *in vivo* [28, 30, 35, 59]. We found that the mice vaccinated with M2e5x protein alone did not induce antibody responses against M2e but showed a higher survival rate compared with that in naïve control mice. Moreover, the frequency of M2e-specific IFN-γ-producing T cells was increased as evidenced by ELISpot and flow cytometry and viral titers were decreased in the lungs of mice vaccinated with M2e5x protein alone compared to those of naïve mice after influenza challenge. These observations suggest that M2e5x protein alone is less immunogenic to induce humoral responses but can elicit M2e-specific cellular immune responses, likely contributing to partial protection.

It was reported that Fc receptors play an important role in conferring M2e specific antibody-mediated protection [29, 35, 37]. However, the possible roles of common γ chain-associated Fc receptors in secreting cytokines by activated macrophages that can opsonize influenza-infected cells with M2e antibody remain unknown. We compared BMDMs from WT and FcR γ^-/-^ mice or cytokine production after incubation with immune complexes of influenza A virus-infected cells. Interestingly, WT BMDM secreted high amounts of TNF-α, IL-6 and IL-12 in response to an immune complex of influenza virus-infected cells and M2e5x.AS04-immunized sera. However, FcR γ^-/-^ BMDM did not produce cytokines in response to M2e-immune complex of influenza virus-infected cells. In particular, IL-12 produced by phagocytic macrophages plays critical roles in the regulation of antigen-presenting cells and effector lymphocytes during the induction of an immune response to pathogens [60]. Moreover, IL-12 has been shown to target naïve, resting CD4^+^ T cells to promote their proliferation and secretion of cytokines. The frequency of M2e-specific IFN-γ-producing T cells was significantly increased in the lungs of mice vaccinated with M2e5x.AS04. These results suggest that the increased levels of IL-12 secreted by phagocytic macrophages engulfing M2e-immune complexes might also contribute to protection against influenza virus. Mouse species are described to have the activating Fc receptors FcγRI, FcγRIII, and FcγRIV and the inhibitory Fc receptor FcγRII [61]. It is also known that mouse IgG1 isotype antibody binds not only to activating FcγRIII but also to inhibitory Fc receptor FcγRII. However, FcγRIII and FcγRIIB are low-affinity receptors for mouse IgG1, IgG2a, and IgG2b [62]. In contrast, FcγRI and FcγRIV are a high-affinity receptor for mouse IgG2a [63]. Since we found that immunization with M2e5x.AS04 induced M2e-specific IgG2a antibodies even at a lower level than IgG1 antibodies, cytokines produced from WT BMDM might be due to IgG2a antibody-mediated binding and opsonizing influenza virus-infected cells via activating FcγRs. Previously, El Bakkouri *et al*. reported that elimination of the common γ chain, which is crucial for intracellular signaling of the activating Fc receptors, diminished protection by M2e-specific IgG [37]. Concordantly, our findings in this study provide evidence that common γ chain-associated Fc receptors play an important role in the production of cytokines by macrophages opsonizing M2e-specific antibody immune complexes of influenza virus-infected cells.

In summary, intramuscular immunization of AS04-adjuvanted M2e5x protein was found to induce broad protective immune responses against divergent M2e epitopes from human, swine, and avian influenza A viruses. In addition, AS04-adjuvanted M2e5x protein immunization was able to generate immunologic memory by rapid recall responses of humoral and cellular immune components upon lethal influenza virus infection. Fc receptors may play an important role in macrophage cell-mediated clearance of virus-infected cells through M2e antibody and in secreting cytokines for inducing protective immune responses, which remains to be tested by further studies. Therefore, results in this study provide evidence that yeast-expressed M2e5x protein could be used as a potential candidate for a broadly protective influenza vaccine.

## Supporting Information

S1 Fig Purification of M2e5x from fed-batch culture broth of *P*. *pastoris*. (A) Flow chart of M2e5x production. (B) Q-sepharose chromatogram of M2e5x.
(TIF)

S2 Fig IFN-γ-producing M2e-specific CD4^+^ and CD8^+^ T cell responses in lungs. IFN-γ–secreting CD4^+^ (A) or CD8^+^ T cells in lungs. Lung cells were harvested, stimulated with M2e peptide, and stained with CD45, CD4, and CD8α surface marker antibodies and intracellularly stained with IFN-γ antibodies, and then analyzed by flow cytometry. Data represent mean ± SEM. Statistical significances were determined by 1-way ANOVA. Asterisks indicate significant differences (**p < 0.01) compared with the results in the naïve group.
(TIF)
