# Supplementary figures and images for: Cross Protection against Influenza A Virus by Yeast-Expressed Heterologous Tandem Repeat M2 Extracellular Proteins
Source: PLoS One. 2015 Sep 14;10(9):e0137822. doi: 10.1371/journal.pone.0137822 (PMC4569442; doi:10.1371/journal.pone.0137822)

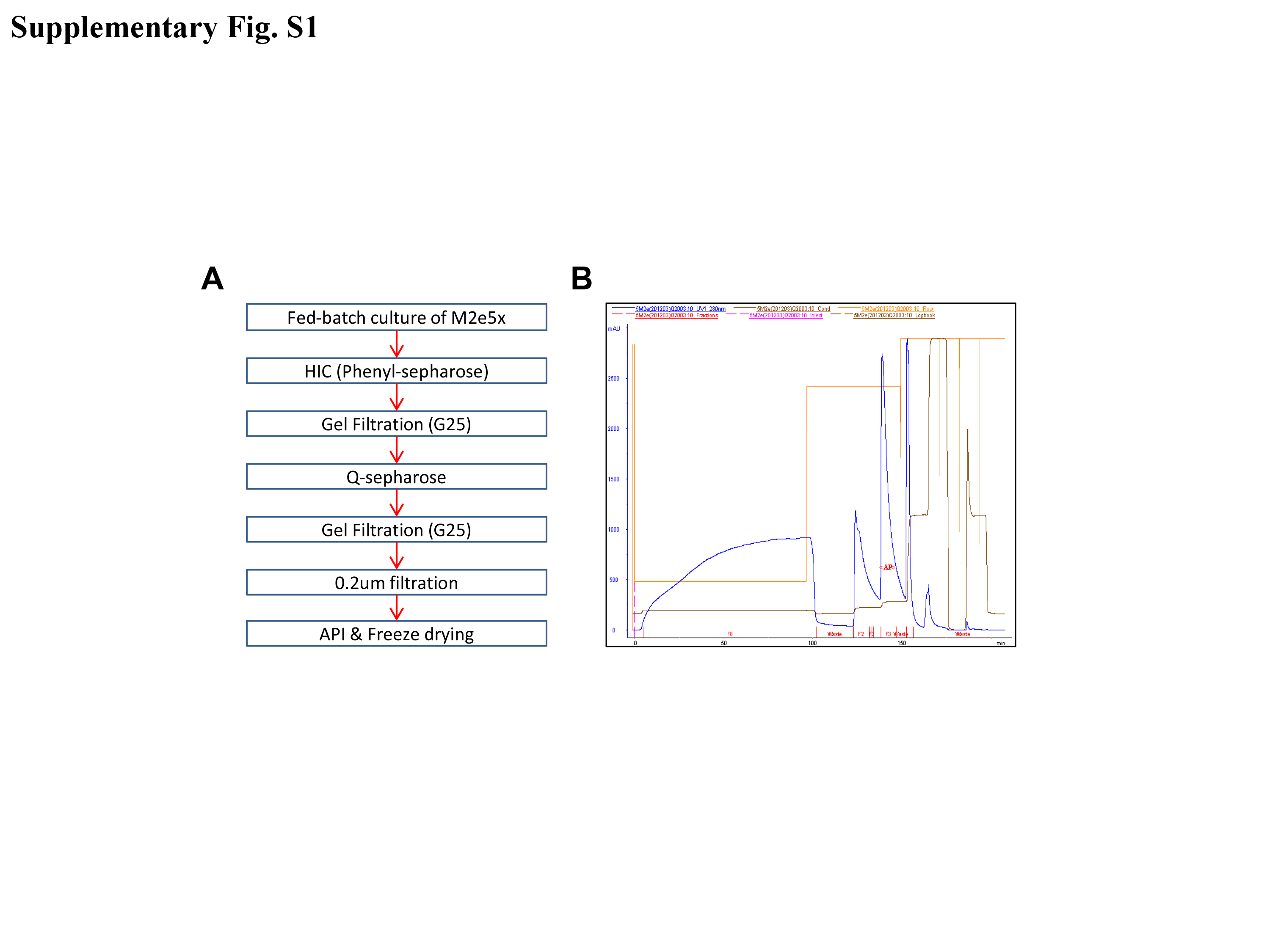

Supplement: S1 Fig — (A) Flow chart of M2e5x production. (B) Q-sepharose chromatogram of M2e5x. (TIF) [file pone.0137822.s001.tif]

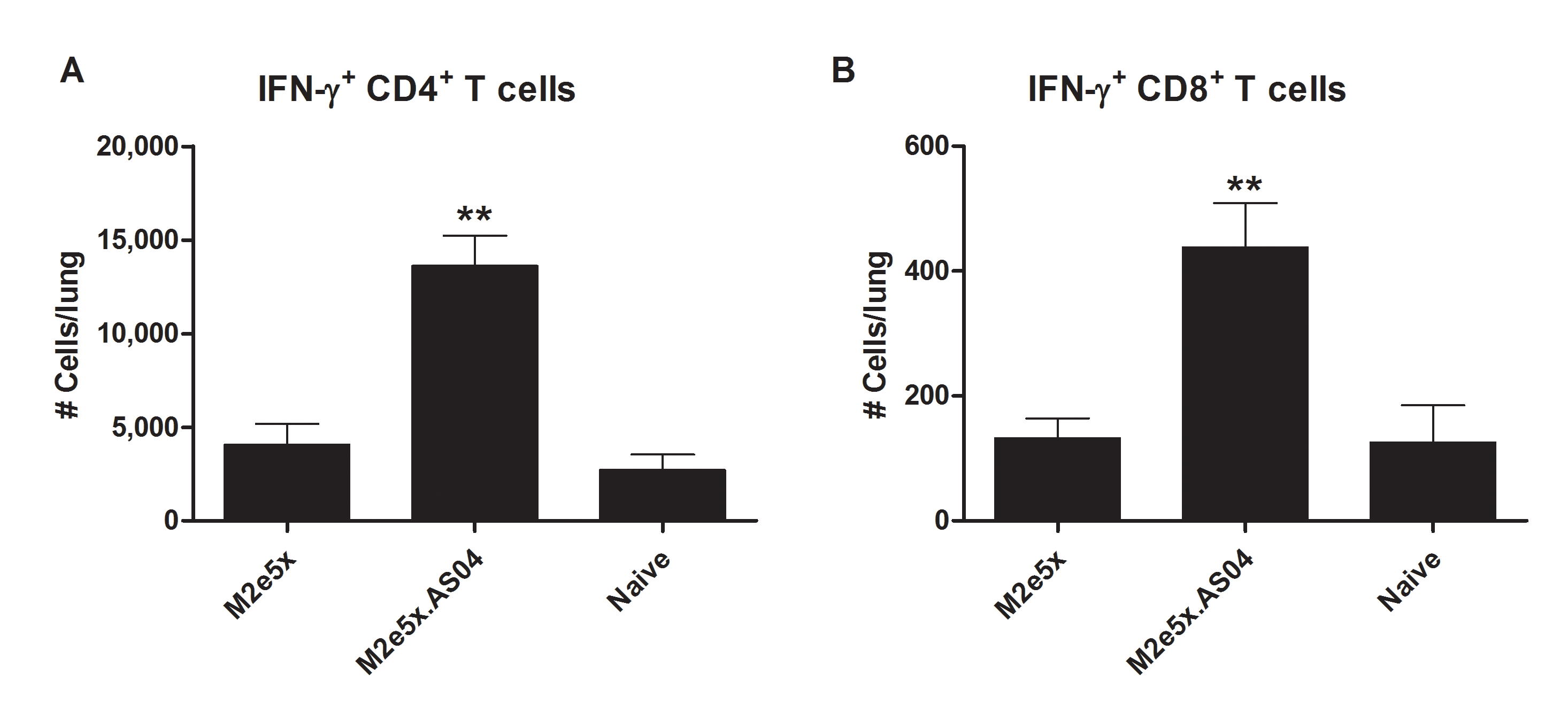

Supplement: S2 Fig — IFN-γ–secreting CD4+ (A) or CD8+ T cells in lungs. Lung cells were harvested, stimulated with M2e peptide, and stained with CD45, CD4, and CD8α surface marker antibodies and intracellularly stained with IFN-γ antibodies, and then analyzed by flow cytometry. Data represent mean ± SEM. Statistical significances were determined by 1-way ANOVA. Asterisks indicate significant differences (**p < 0.01) compared with the results in the naïve group. (TIF) [file pone.0137822.s002.tif]
